# Supplementary material for: Predicting visual function by interpreting a neuronal wiring diagram
Source: Nature. 2024 Oct 2;634(8032):113–23. doi: 10.1038/s41586-024-07953-5 (PMC11446822; doi:10.1038/s41586-024-07953-5)
Supplement: Supplementary file 6 — CRF and ERF predictions for individual TmY4 and TmY9 cells. Analogous to Supplementary Data 3, but for TmY target types. Shown are the top four monosynaptic pathways, the strongest pathway passing through each of the top ten intermediary types (ranking from Extended Data Fig. 7), and the trisynaptic pathway Tm1–TmY–Dm3–TmY (see the section entitled Prediction of spatial normalization). [file 41586_2024_7953_MOESM6_ESM.zip › DataS4/TmY9q/720575940618756893.pdf]

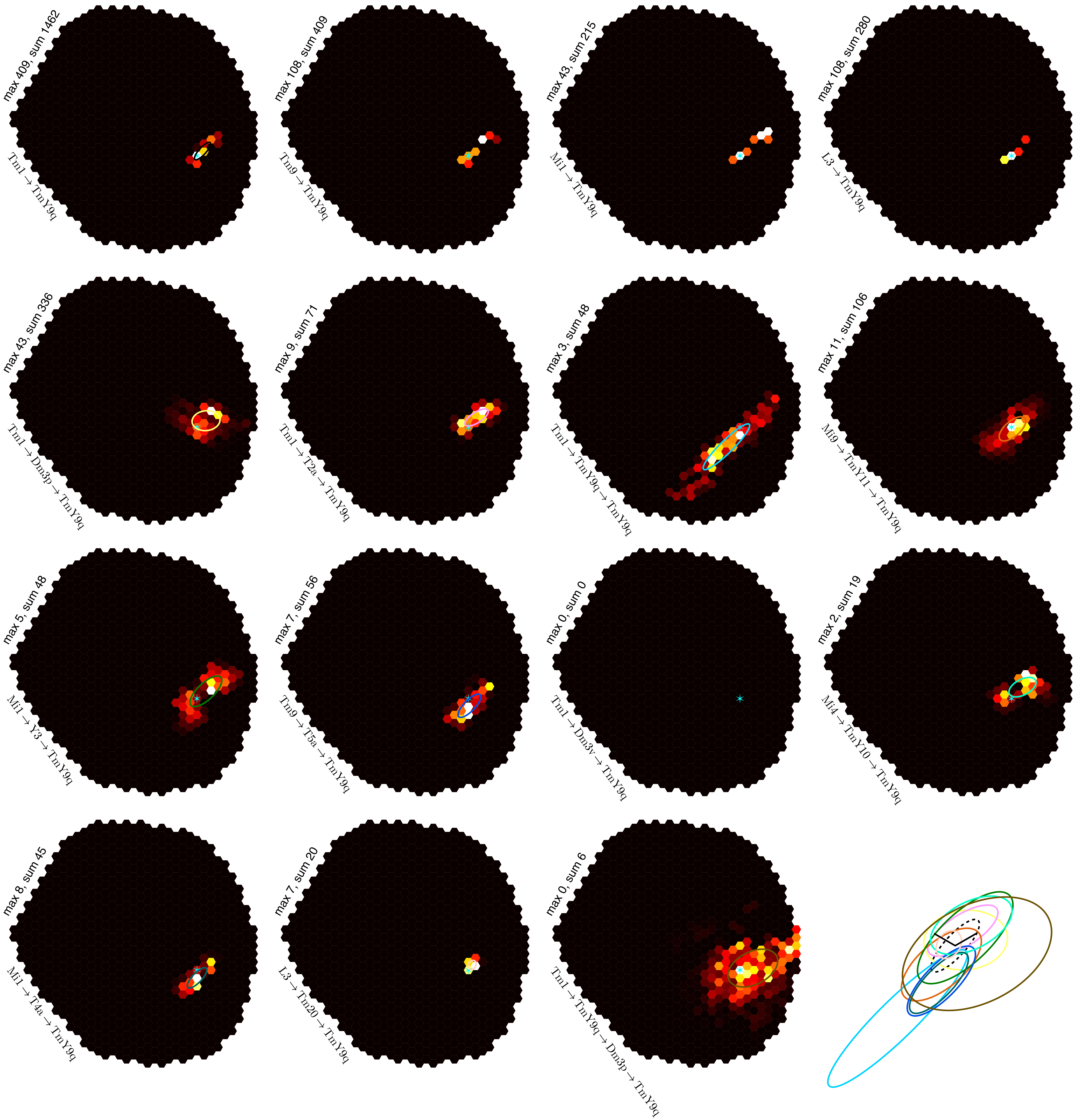

max 409, sum 1462
Tm1 → TmY9q
max 108, sum 409
Tm9 → TmY9q
max 43, sum 215
Mi1 → TmY9q
max 108, sum 280
L3 → TmY9q
max 43, sum 336
Tm1 → Dm3p → TmY9q
max 9, sum 71
Tm1 → T2a → TmY9q
max 3, sum 48
Tm1 → TmY9q → TmY9q
max 11, sum 106
Mi9 → TmY11 → TmY9q
max 5, sum 48
Mi1 → Y3 → TmY9q
max 7, sum 56
Tm9 → T5a → TmY9q
max 0, sum 0
Tm1 → Dm3v → TmY9q
max 2, sum 19
Mi4 → TmY10 → TmY9q
max 8, sum 45
Mi1 → T4a → TmY9q
max 7, sum 20
L3 → Tm20 → TmY9q
max 0, sum 6
Tm1 → TmY9q → Dm3p → TmY9q
